# Supplementary material for: Discrete subicular circuits control generalization of hippocampal seizures
Source: Nat Commun. 2022 Aug 25;13:5010. doi: 10.1038/s41467-022-32742-x (PMC9411516; doi:10.1038/s41467-022-32742-x)
Supplement: Supplementary file 1 — Supplementary Information [file 41467_2022_32742_MOESM1_ESM.pdf]

Supplementary information for

Discrete Subicular Circuits Control Generalization of Hippocampal Seizures

Fan Fei[1, a], Xia Wang[1, a], Cenglin Xu[2, a*], Jiaying Shi[1], Yiwei Gong[1, 2], Heming Cheng[2], Nanxi Lai[1], Yeping Ruan[2], Yao Ding[3], Shuang Wang[3], Zhong Chen[1, 2, 3*] and Yi Wang[1, 2, 3*]

[1] Institute of Pharmacology and Toxicology, College of Pharmaceutical Sciences, Zhejiang University, Hangzhou, China;

[2] Key Laboratory of Neuropharmacology and Translational Medicine of Zhejiang Province, School of Pharmaceutical Sciences, Zhejiang Chinese Medical University, Hangzhou, China;

[3] Epilepsy Center, Second Affiliated Hospital, School of Medicine, Zhejiang University, Hangzhou, Zhejiang, China

[a] These authors contributed equally to this paper.

*Correspondence to:

Professor Yi Wang (wang-yi@zju.edu.cn), Professor Zhong Chen (chenzhong@zju.edu.cn), Prof. Cenglin Xu (xucenglin5zz@zju.edu.cn).

Tel & Fax: +86-571-88208228

This PDF file includes:

Figures. S1 to S14

Table. S1 and S2

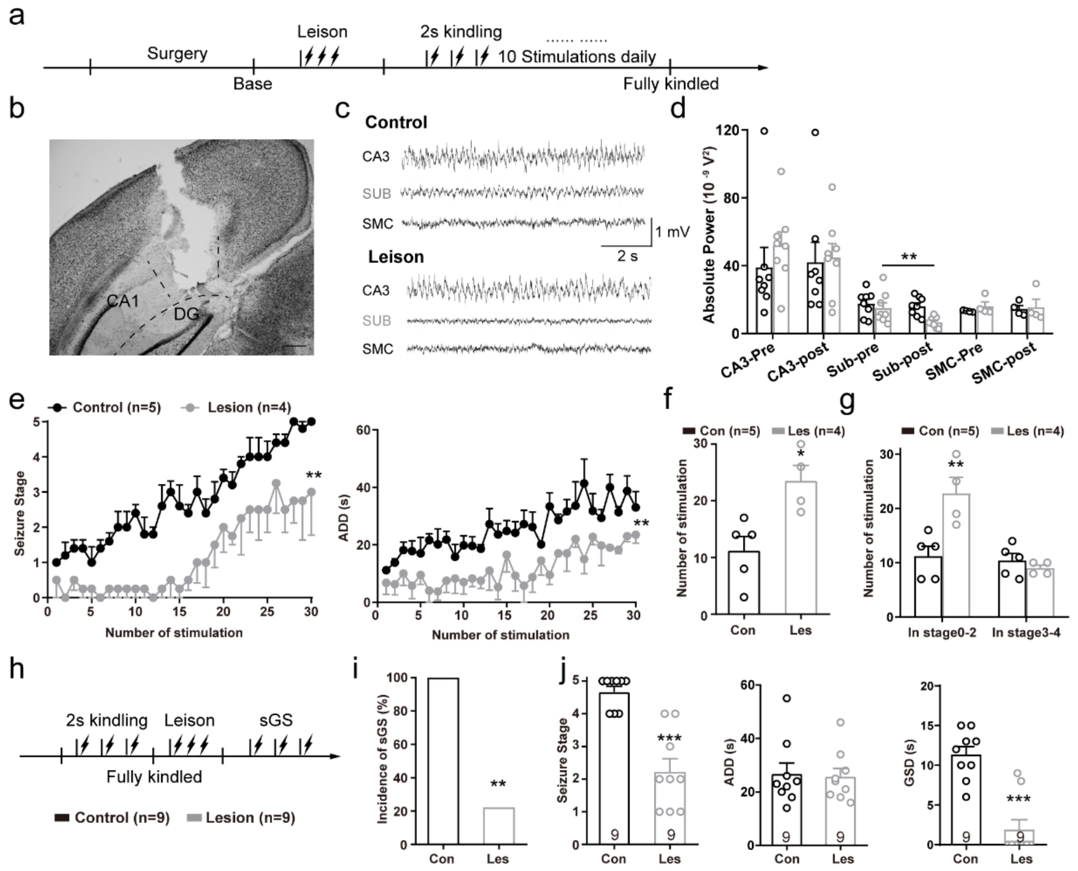

Figure S1 Lesions in the subiculum suppress hippocampal seizures. a Scheme of lesion experiments of the subiculum in secondary generalized seizure (sGS) acquisition of hippocampal kindling model. For electrical lesion studies, we used 1 mA, 10 s, direct current stimulation in the subiculum. **b** Representative image of electronic lesion in the subiculum. Scale bar, 200 μm. **c** Representative EEGs of hippocampal CA3, SUB, and secondary motor cortex (SMC) between control and lesion group. **d** Graph of absolute power of CA3, SUB, and SMC in both control and lesion group. Paired *t*-test, $^{**}P<0.01$. Two channels for CA3 and SUB recording, one channel for SMC recording in each mouse (N = 4 for each group). **e** Effects of lesion of the subiculum on the development of seizure stage and after-discharge durations (ADD). Two-way repeated measures ANOVA, $^{**}P<0.01$. **f** Number of stimulations needed to reach sGS. Unpaired *t*-test, $^{*}P<0.05$. **g** Number of stimulations spent in stages 0-2 and stages 3-4. Unpaired *t*-test, $^{**}P<0.01$. **h** Scheme of lesion experiments of the subiculum in sGS expression of hippocampal kindling model. **i** Effects of lesions in the subiculum on the incidence of sGS (N = 9 for each group). Fisher's exact test, $^{**}P<0.01$. **j** Effects of lesions of the subiculum on seizure stage, ADD, and GS duration (GSD) during sGS expression. Unpaired *t*-test, $^{***}P<0.001$. The number of mice used in each group is indicated in figure. Data are shown as means ± SEM.

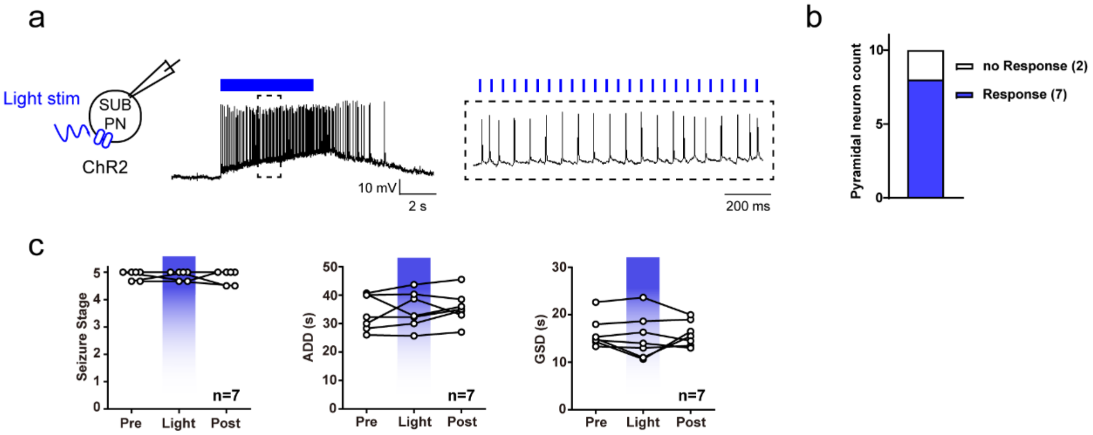

Figure S2 Functional validation of ChR2-expressed subicular pyramidal neurons and effects of light on hippocampal seizures in mice without ChR2. a Left, scheme of 473 nm light stimulation and patch in the subicular pyramidal neurons expressed ChR2. Right, representative APs recording from a ChR2-expressing subicular pyramidal neuron with light stimulation (473 nm, 20Hz, 10ms/pulse, 5s). The blue bar indicated light stimulation period. **b** Number of responded neurons in respond to light stimulation (7/9 neurons from 3 mice). **c** Effects of 473 nm light stimulation of subicular pyramidal neurons on the seizure stage, after-discharge duration (ADD) and generalized seizure duration (GSD) during sGS expression in mice without ChR2. The number of mice used in each group is indicated in figure. Data are presented as means ± SEM.

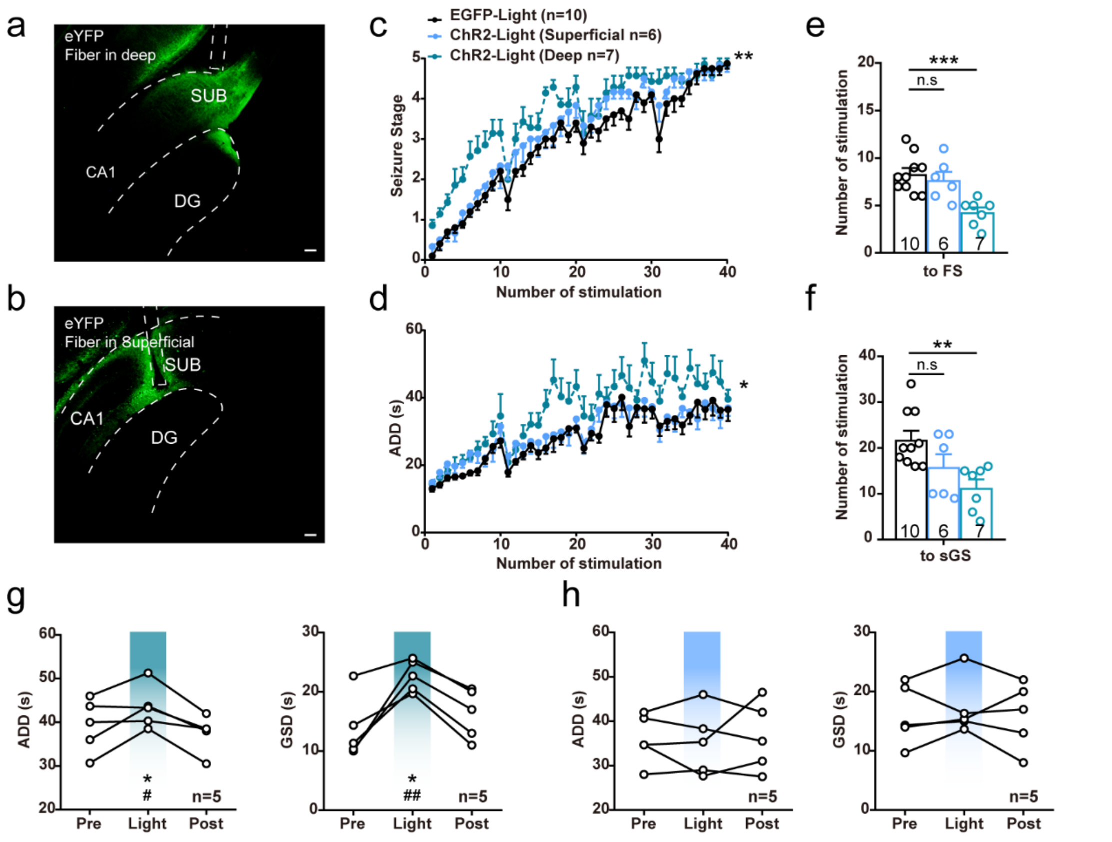

Figure S3 Optogenetic activation of subicular pyramidal neurons in the deep layer, rather than the superficial layer, accelerates the generalization of hippocampal seizures. a, b Representative images of cannula placement and ChR2 expression in the deep (**a**) and superficial (**b**) layers of the subiculum. Scale bar, 100 μm. **c, d** Effects of optogenetic activation of deep and superficial subicular pyramidal neurons on the development of seizure stage (**c**) and after-discharge duration (ADD, **d**). Two-way repeated measures ANOVA with *post hoc* Scheffe's test, **P*<0.05, ***P*<0.01 ChR2-Light (deep) compared to EGFP-Light group. **e, f** Number of stimulations needed to reach FS (stage 2, **e**) and sGS (**f**). One-way ANOVA with *post hoc* Dunnett's test, ***P*<0.01 compared to EGFP-Light group, ****P*<0.001 compared to EGFP-Light group. **g-h** Effects of optogenetic activation of the deep (**g**) and superficial (**h**) layer of subicular pyramidal neurons on the ADD and GS duration (GSD) during sGS expression. One-way repeated measures ANOVA with *post hoc* Dunnett's test, **P*<0.05 compared to Pre, #*P*<0.05 compared to Post, ##*P*<0.01 compared to Post. The number of mice used in each group is indicated in figure. Data are presented as means ± SEM.

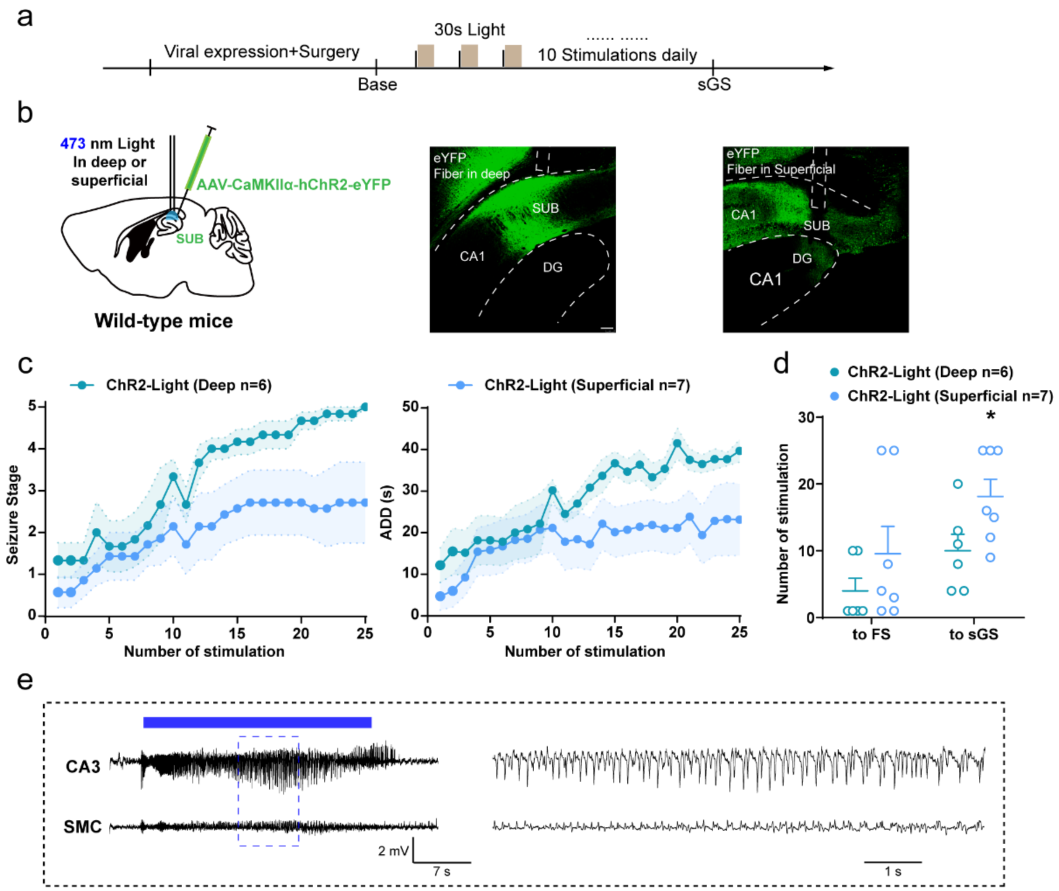

Figure S4 Repetitive activation of subicular pyramidal neurons induces kindling-like seizure generalization. a Scheme of experiments for optogenetic activation of subicular pyramidal neurons. **b** Representative images of wild-type mice injected with AAV-CaMKIIα-ChR2-eYFP and optical cannula placements in the deep and superficial subiculum. Scale bar, 100 μm. **c** Effects of optogenetic activation of deep and superficial layer of subicular pyramidal neurons on the development of seizure stage and after-discharge duration (ADD). **d** Number of stimulations needed to reach FS (stage 2) and secondary generalized seizure (sGS) between ChR2-Light (deep) and ChR2-Light (superficial) groups. Mice that could not develop into seizures with 25 times stimulation were calculated as 25. Mann-Whitney test, **P*<0.05. **e** Typical EEGs recorded from the hippocampal CA3 and secondary motor cortex (SMC) in a mouse during light-induced sGS. The blue bar indicated the period of light stimulation. The number of mice used in each group is indicated in figure. Data are presented as means ± SEM.

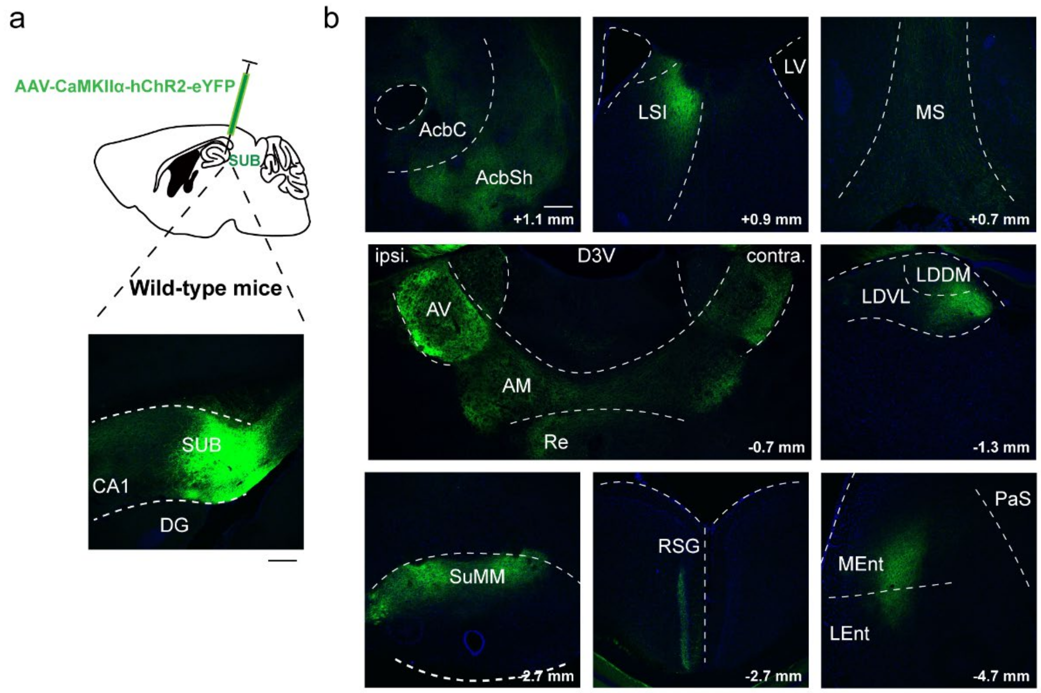

Figure S5 Long-range output organization of subicular pyramidal neurons. a Scheme of experiments for viral expression of ChR2-eYFP in the SUB. Scale bar, 200 μm, also applies to **b**. **b** eYFP florescence highlighted the downstream areas of subicular projecting pyramidal neurons in mice. Numbers in the bottom right indicated the approximate distance to the bregma. AcbC, accumbens nucleus, core; AcbSh, accumbens nucleus, shell; LSI, lateral septum, intermediate part; LV, lateral ventricle; MS, medial septum; D3V, dorsal 3rd ventricle; AV, anteroventral thalamic nucleus; AM, anteromedial thalamic nucleus; Re, reuniens thalamic nucleus; LDDM, laterodorsal thalamic nucleus; LDVL, ventrolateral thalamic nucleus; SuMM, supramammillary nucleus, medial part; RSG, retrosplenial granular cortex; PaS, parasubiculum; MEnt, medial entorhinal cortex; LEnt, lateral entorhinal cortex.

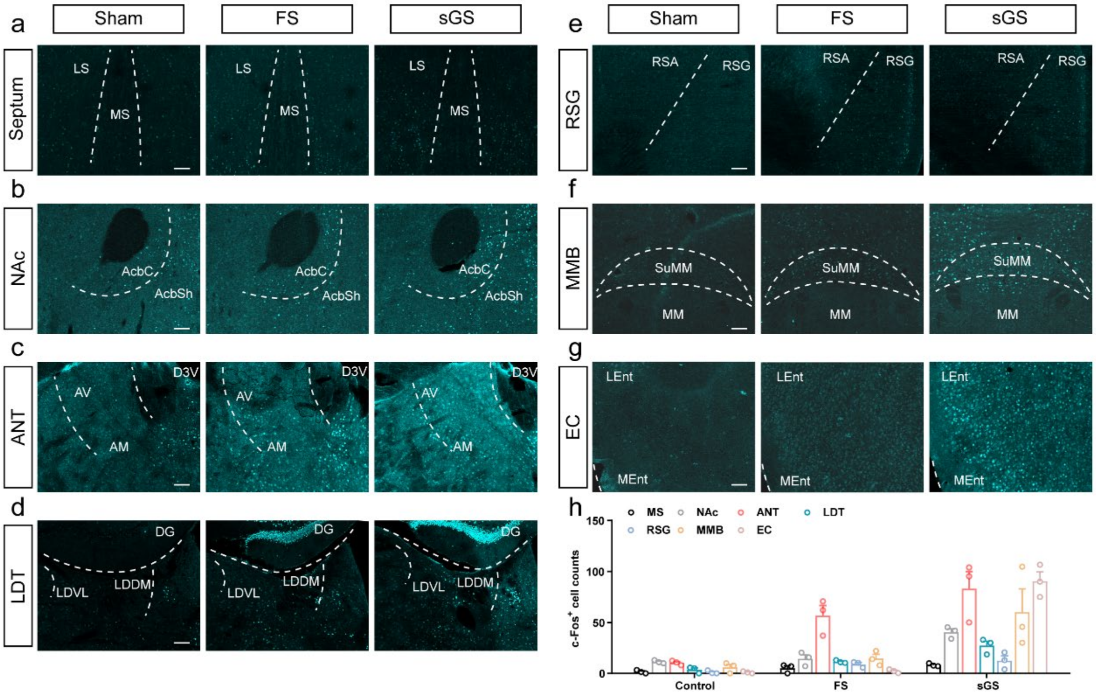

Figure S6 Distinct c-Fos activation patterns in multiple downstream areas of the subiculum after seizures. a-g Representative c-Fos activation patterns in mice without seizures (Sham), or mice that only underwent focal seizure (FS) or secondary generalized seizure (sGS) in several brain regions, including the septum (**a**), nucleus accumbens (NAc, **b**), anterior nucleus of thalamus (ANT, **c**), laterodorsal thalamic nucleus (LDT, **d**) retrosplenial granular cortex (RSG, **e**), mammillary bodies (MMB, **f**), and the entorhinal cortex (EC, **g**). Scale bar, 100 µm. LS, lateral septum. MS, medial septum. AcbC, accumbens nucleus core. AcbSh, accumbens nucleus shell. D3V, dorsal 3rd ventricle. AV, anteroventral thalamic nucleus. AM, anteromedial thalamic nucleus. LDDM, laterodorsal thalamic nucleus; LDVL, ventrolateral thalamic nucleus. RSG, retrosplenial granular cortex. SuMM, supramammillary nucleus. MM, medial mammillary nucleus. LEnt, lateral entorhinal cortex. MEnt, medial entorhinal cortex. **h** Quantification of total activated c-Fos after different seizure stage in brain areas of **a-g** (N = 3 mice, each 3 slices for each group). Data are presented as means ± SEM.

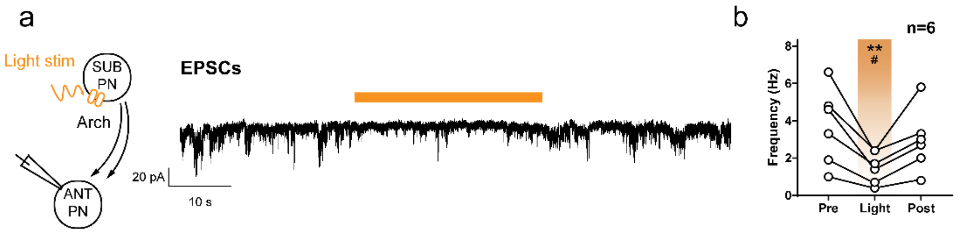

Figure S7 Functional validation of Arch-expressed subicular projecting terminals in the ANT. a Left, scheme of 589 nm light stimulation in the subicular pyramidal neurons expressed with Arch and patch recording in the ANT pyramidal neurons. Right, representative EPSC recording from an ANT neuron during light stimulation (30 s, DC). The yellow bar indicated light stimulation period. **b** Effects of optogenetic hyperpolarization of subicular pyramidal neurons on EPSCs recording from ANT neurons (N = 6 neurons from 3 mice). Friedman with *post hoc* Dunn's test, ***P*<0.01 Light compared to Pre, #*P*<0.05 Light compared to Post. Data are presented as scattered dots.

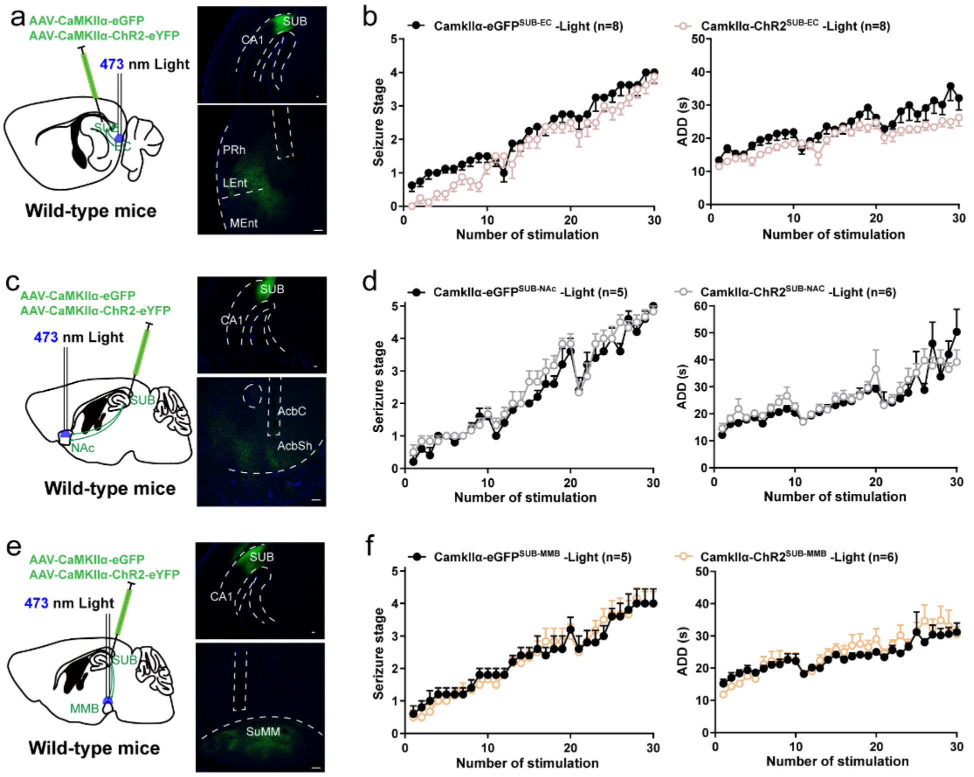

Figure S8 Effects of optogenetic activation of SUB-EC, SUB-NAc and SUB-MMB circuits on hippocampal kindling model. a, c and **e** Left, Scheme of experiments for viral expression of ChR2-eYFP in the subiculum (SUB) and light stimulation in the entorhinal cortex (EC, **a**), nucleus accumbens (NAc, **c**) or mammillary bodies (MMB, **e**) of wild-type mice. Right, corresponding representative optical cannula placements. Scale bar, 100 μm. **b, d** and **f** Effects of optogenetic activation of SUB-EC (**b**), SUB-NAc (**d**) and SUB-MMB (**f**) glutamatergic circuits on the development of seizure stage and after-discharge duration (ADD). The number of mice used in each group is indicated in figure. Data are presented as means ± SEM.

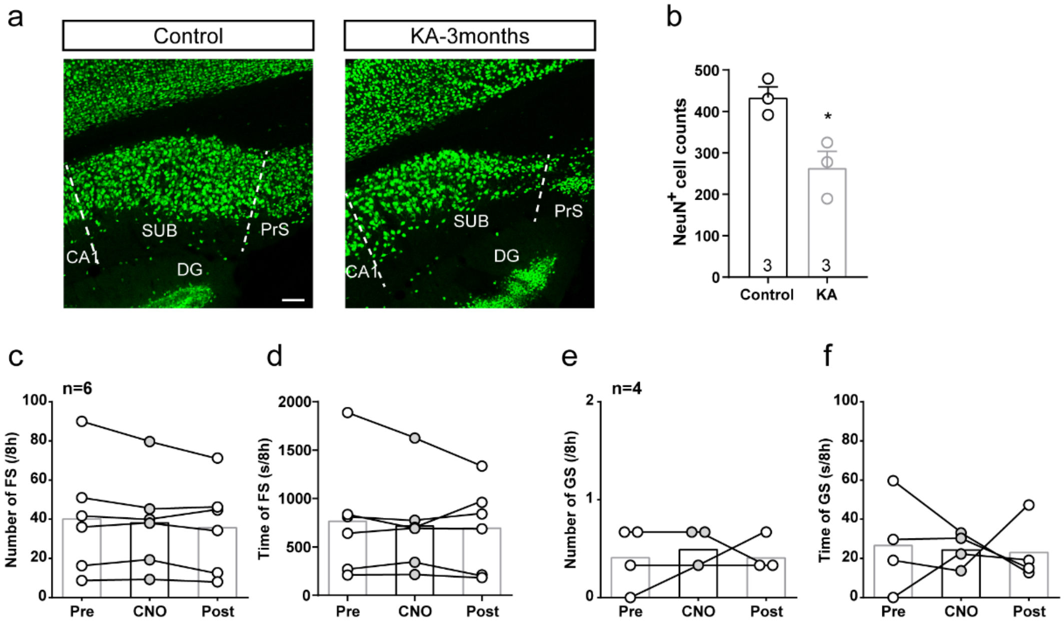

Figure S9 Subicular neuronal loss in KA model and effects of CNO itself on KA-induced seizures in mice without hM4Di. a Representative immunohistochemical images of NeuN in the subiculum of mice without KA injection and 3 months after KA injection. Note an obvious decrease in the number of $NeuN^+$ cells after KA-induced seizures. Scale bar, 100 µm. **b** Quantification of $NeuN^+$ cells in the subiculum of mice in KA model (N = 3 mice for both groups, 3 slices from each mouse). Unpaired t test, *$P<0.05$. **c-f** Effects of CNO injection in the ANT of *CaMKIIα*-mCherry ^{SUB} mice on the number and duration of FSs (**c**, **d**) and sGSs (**e**, **f**). The number of mice used in each group is indicated in figure. Data are presented as means ± SEM.

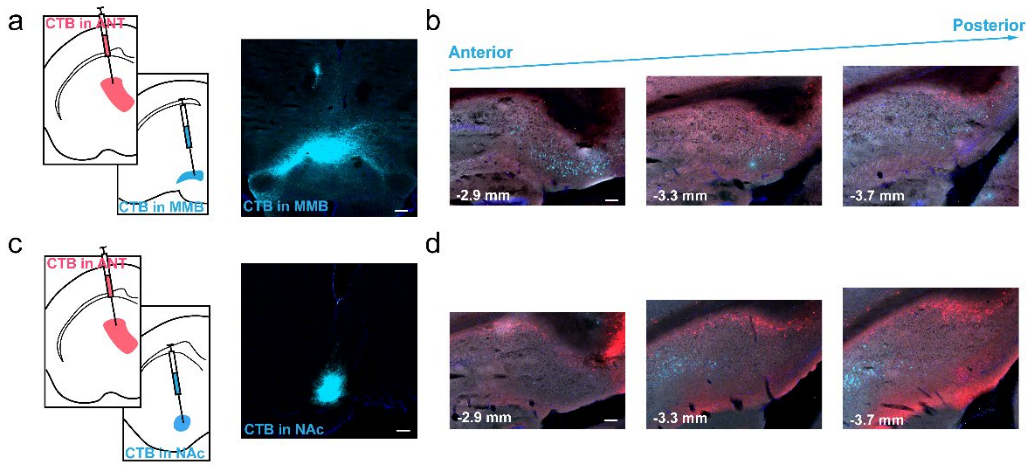

Figure S10 Somata locations of the ANT-projecting subicular neurons are different from those of the MMB-projecting and NAc-projecting neurons in the subiculum. a Left, scheme of CTB injection in the ANT and MMB. Scale bar, 100 μm, also applies to **b-d**. **b** Representative images of CTB somata location from the ANT and MMB in the subiculum from the anterior to posterior axis. Numbers in the bottom left indicated the approximate distance to the bregma, the same in **d**. **c** Left, scheme of CTB injection in the ANT and NAc. **d** Representative images of CTB somata location from the ANT and NAc in the subiculum from the anterior to posterior axis.

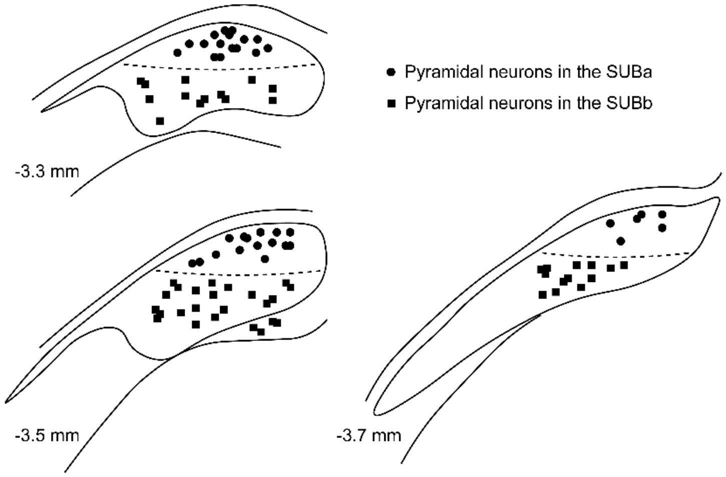

Figure S11 Electrode placements for *in vivo* multi-unit recordings in the subiculum. Circles represented recording neurons in the SUBa, and squares represented recording neurons in the SUBb. Numbers in the figure indicated the approximate distance to the bregma. N = 87 units from 14 mice.

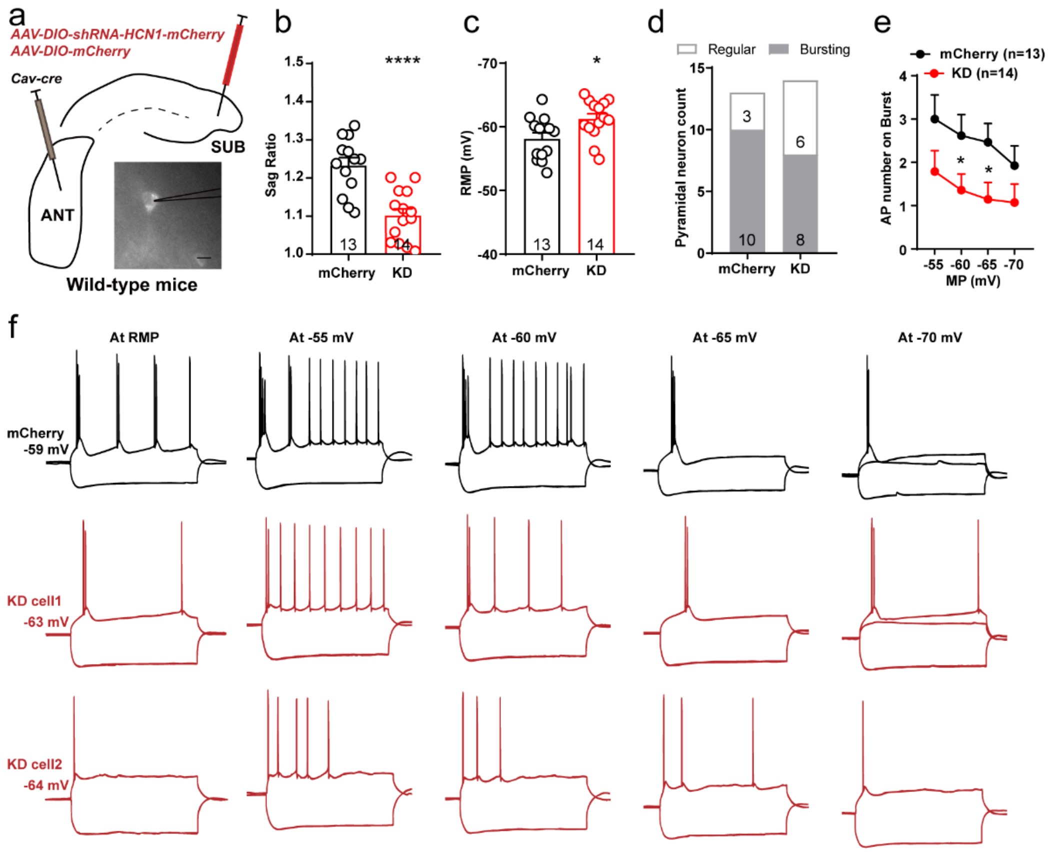

Figure S12 Effect of conditional HCN knockdown on ANT-projecting subicular pyramidal neuron on bursting firing. a Left, scheme of experiments for viral expression in the ANT and SUB for conditional knockdown of HCN expression in ANT-projecting subicular neurons of wild-type mice. Right, representative image of patch clamp in a subicular pyramidal neuron. Scale bar, 10 μm. **b-d** Graph of sag ratio (**b**), rest membrane potential (RMP, **c**) and the number of bursting/regular spiking neurons (**d**) in pyramidal neurons of mCherry (n = 13 neurons from 4 mice, 10 bursting) and ShRNA-HCN1$^{ANT\text{-}SUB}$ (KD) mice (n = 14 neurons from 3 mice, 8 bursting). Unpaired t-test for sag ratio and RMP, ****P<0.0001, *P<0.05. **e** Graph of AP numbers on burst by 100-pA injection at -55 mV, -60 mV, -65 mV and -70 mV membrane potential (MP). Mann-Whitney test, *P<0.05. **f** Representative action potentials in neurons evoked by -200-pA and 100-pA (150-pA was also showed for mCherry and KD cell1 at -70 mV to elicit AP firing) injection current of mCherry and KD. The number of mice used in each group is indicated in figure. Data are presented as means ± SEM.

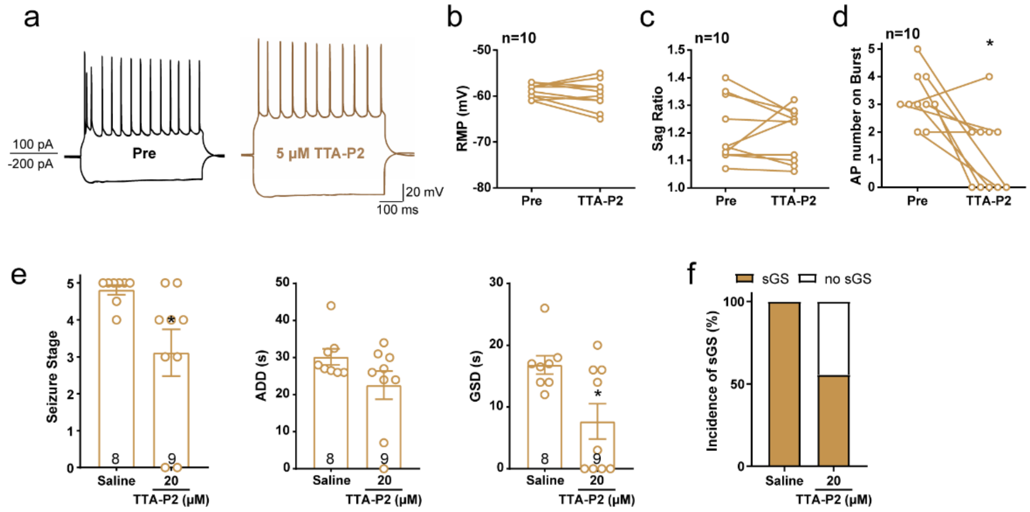

Figure S13 Effect of T-type calcium channels blocker on bursting firing of subicular pyramidal neuron and hippocampal seizures. a Representative action potential in one subicular bursting neuron before and after incubation of 5 μM T-type calcium channel blocker, TTA-P2. **b-d** Graph of rest membrane potential (**b**), sag ratio (**c**) and action potential number on burst (**d**) before and after TTA-P2 incubation. Wilcoxon matched-pairs signed rank test, **P*<0.05 in AP numbers on burst, no significance in RMP and sag ratio (n = 10 neurons from 4 mice). **e** Effects of TTA-P2 (intra-subicular injection, 500 nL) on the seizure stage, after-discharges duration (ADD), and generalized seizure duration (GSD) of sGS expression. Mann-Whitney test, **P*<0.05 compared with Saline group. **f** Effects of TTA-P2 on the incidence of sGS. The number of mice used in each group is indicated in figure. Data are presented as means ± SEM.

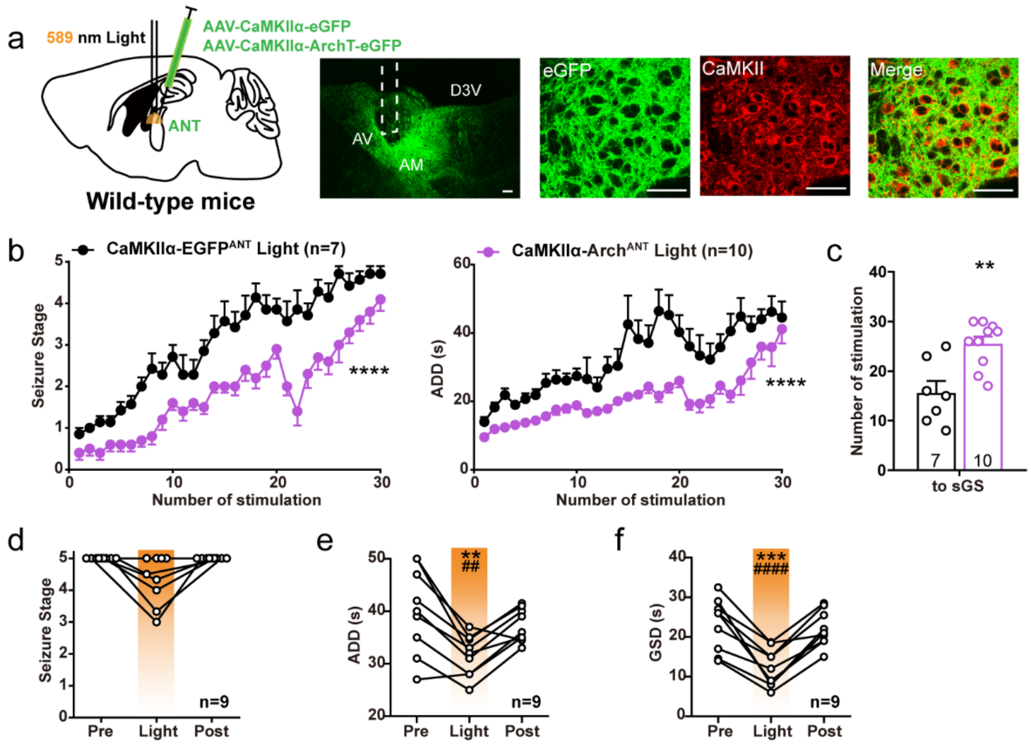

Figure S14 Optogenetic hyperpolarization of ANT glutamatergic neurons retards hippocampal seizures. a Left, scheme of experiments for viral expression and light stimulation in the ANT of wild-type mice. Right, representative images of the optical cannula placement and Arch expression in the ANT. Scale bar, 50 μm. **b** Effects of optogenetic hyperpolarization of ANT glutamatergic neurons on the development of seizure stage and after-discharge duration (ADD). Two-way repeated measures ANOVA, ****P<0.0001. **c** Number of stimulations needed to reach sGS. Unpaired t-test, **P<0.01. **d-f** Effects of optogenetic hyperpolarization of ANT glutamatergic neurons on seizure stage, ADD, and generalized seizure duration (GSD) during sGS expression. One-way repeated measures ANOVA with *post hoc* Dunnett's test, **P<0.01, ***P<0.001 compared to Pre, ##P<0.01, ####P<0.0001 compared to Post. The number of mice used in each group is indicated in figure. Data are presented as means ± SEM.

Table S1 *In vitro* Electrophysiological Measurements of the ANT-Projecting and non-ANT-Projecting Subicular Pyramidal Neurons

Property	ANT-projecting Bursting	ANT-projecting Regular	Non-ANT-projecting Bursting	Non-ANT-projecting Regular
**Membrane Potential (mV)**	-60.34 ± 2.05	-63.13 ± 3.35	-60.46 ± 1.51	-54.47 ± 2.54
**Sag Ratio**	1.31 ± 0.022	1.22 ± 0.025*	1.17 ± 0.015****	1.17 ± 0.029***
**Input resistance (MΩ)**	211.67 ± 18.02	235.17 ± 24.10	191.44 ± 21.15	243.58 ± 29.89
**Time constant (ms)**	12.79 ± 0.74	10.51 ± 1.06	9.59 ± 0.84	13.46 ± 1.30
**Rheobase (pA)**	26.88 ± 6.17	34.44 ± 5.56	43.75 ± 5.45	28.63 ± 6.33
**AP amplitude (pA)**	87.05 ± 3.03	81.85 ± 2.67	91.95 ± 2.08	80.73 ± 3.03
**Half-width (ms)**	1.11 ± 0.08	1.41 ± 0.21	1.48 ± 0.14	1.51 ± 0.17
**AP numbers on burst**	4.13 ± 0.27		3.19 ± 0.18**	
**AP intervals on burst (ms)**	11.29 ± 0.55		13.18 ± 0.73*	

One-way ANOVA with *post hoc* Dunnett's test for multiple comparisons. **P* < 0.05, ***P* < 0.01, ****P* < 0.001, *****P* < 0.0001, compared with data in the first column.

Table S2 Statistical summary for main figures and supplementary figures

Figure	Group	Sample size	Test used	Treatment effect	P-value
**1e**	c-Fos$^+$ cell count	N=4, 3, 5 mice	One-way ANOVA	F (2, 9) = 29.12	
	Sham vs. FS		*Post hoc* Dunnett's		0.0255
	Sham vs. sGS		*Post hoc* Dunnett's		<0.0001
**1g**	Dis vs. Pro	N=5, 5 mice	Unpaired t	Df = 4, t=1.51	0.2055
	Dee vs. Sup	N=5, 5 mice	Unpaired t	Df = 4, t=3.531	0.0242
**1i**	Seizure stage	N=10, 8, 13 mice	Two-way RM ANOVA	F (2, 28) = 13.74	
	eGFP vs. ChR2		*Post hoc* Scheffe's		0.0176
	eGFP vs. Arch		*Post hoc* Scheffe's		0.0459
	ADD	N=10, 8, 13 mice	Two-way RM ANOVA	F (2, 28) = 8.396	
	eGFP vs. ChR2		*Post hoc* Scheffe's		0.0125
	eGFP vs. Arch		*Post hoc* Scheffe's		0.0103
**1j**	To FS	N=10, 8, 13 mice	One-way ANOVA	F (2, 28) = 11.22	
	eGFP vs. ChR2		*Post hoc* Dunnett's		0.0255
	eGFP vs. Arch		*Post hoc* Dunnett's		0.0824
**1k**	To sGS	N=10, 8, 13 mice	One-way ANOVA	F (2, 28) = 14.81	
	eGFP vs. ChR2		*Post hoc* Dunnett's		0.0097
	eGFP vs. Arch		*Post hoc* Dunnett's		0.0443
**1p**	GSD	N = 10 mice	One-way RM ANOVA	F (9, 18) = 3.714	
	Pre vs. Light		*Post hoc* Dunnett's		0.0396
	Post vs. Light		*Post hoc* Dunnett's		0.0265
**1s**	GSD	N = 6 mice	One-way RM ANOVA	F (5, 10) = 3.630	
	Pre vs. Light		*Post hoc* Dunnett's		0.0087
	Post vs. Light		*Post hoc* Dunnett's		0.0015
**2b**	SS EGFP vs. ChR2	N = 10, 12 mice	Two-way RM ANOVA	F (1, 20) = 13.96	0.0013
	ADD EGFP vs. ChR2		Two-way RM ANOVA	F (1, 20) = 17.28	0.0005
**2c**	EGFP vs. ChR2	N = 10, 12 mice	Unpaired t	Df = 20, t = 2.272	0.0343
**2e**	ADD	N = 11 mice	One-way RM ANOVA	F (10, 20) = 4.895	
	Pre vs. Light		*Post hoc* Dunnett's		0.0352
	Post vs. Light		*Post hoc* Dunnett's		0.0153
**2f**	GSD	N = 11 mice	One-way RM ANOVA	F (10, 20) = 1.875	
	Pre vs. Light		*Post hoc* Dunnett's		0.0065
	Post vs. Light		*Post hoc* Dunnett's		0.0005
**2g**	Pre vs. Light	N = 11 EEG sections	Paired t	Df = 10, t = 2.551	0.0288

**2j**	SS EGFP vs. Arch	N = 9, 11 mice	Two-way RM ANOVA	F (1, 18) = 27.48	<0.0001
	ADD EGFP vs. Arch		Two-way RM ANOVA	F (1, 18) = 8.975	0.0078
**2k**	EGFP vs. Arch	N = 9, 11 mice	Unpaired t	Df = 18, t = 4.905	0.0001
**2l**	SS	N = 10 mice	Friedman	Friedman statistic = 18.82	
	Pre vs. Light		*Post hoc* Dunn's		0.0019
	Post vs. Light		*Post hoc* Dunn's		0.0004
**2m**	ADD	N = 10 mice	One-way RM ANOVA	F (9, 18) = 1.312	
	Pre vs. Light		*Post hoc* Dunnett's		0.0376
	Post vs. Light		*Post hoc* Dunnett's		0.006
**2n**	GSD	N = 10 mice	One-way RM ANOVA	F (9, 18) = 1.897	
	Pre vs. Light		*Post hoc* Dunnett's		0.0079
	Post vs. Light		*Post hoc* Dunnett's		0.0019
**2o**	Pre vs. Light	N = 10 EEG sections	Paired t	Df = 9, t = 3.176	0.0113
**3b**	SS EGFP vs. Arch	N = 9, 11 mice	Two-way RM ANOVA	F (1, 18) = 5.148	0.0358
	ADD EGFP vs. Arch	N = 9, 11 mice	Two-way RM ANOVA	F (1, 18) = 5.029	0.0378
**3c**	ADD	N = 9 mice	One-way RM ANOVA	F (8, 16) = 6.274	
	Pre vs. Light		*Post hoc* Dunnett's		0.0012
	Post vs. Light		*Post hoc* Dunnett's		0.476
**3d**	GSD	N = 9 mice	One-way RM ANOVA	F (8, 16) = 2.022	
	Pre vs. Light		*Post hoc* Dunnett's		0.0197
	Post vs. Light		*Post hoc* Dunnett's		0.5242
**4d**	Number of FS	N = 8 mice	One-way RM ANOVA	F (7, 14) = 5.625	
	CNO vs. Pre		*Post hoc* Dunnett's		0.0014
	CNO vs. Post		*Post hoc* Dunnett's		0.0099
**4e**	Time of FS	N = 8 mice	One-way RM ANOVA	F (7, 14) = 3.090	
	CNO vs. Pre		*Post hoc* Dunnett's		0.0024
	CNO vs. Post		*Post hoc* Dunnett's		0.0133
**4f**	Number of GS	N = 4 mice	Friedman	Friedman statistic = 6.615	
	CNO vs. Pre		*Post hoc* Dunn's		0.0431
	CNO vs. Post		*Post hoc* Dunn's		0.3146
**4g**	Time of GS	N = 4 mice	Friedman	Friedman statistic = 7.6	
	CNO vs. Pre		*Post hoc* Dunn's		0.016
	CNO vs. Post		*Post hoc* Dunn's		0.5777

**4i**	Number of FS	N = 5 mice	One-way RM ANOVA	F (4, 8) = 20.75	
	CNO vs. Pre		*Post hoc* Dunnett's		0.0357
	CNO vs. Post	Excluded the dead	*Post hoc* Dunnett's		0.068
**4j**	Time of FS	N = 5 mice	One-way RM ANOVA	F (4, 8) = 18.97	
	CNO vs. Pre		*Post hoc* Dunnett's		0.034
	CNO vs. Post	Excluded the dead	*Post hoc* Dunnett's		0.0729
**4k**	Number of GS	N = 4 mice	Friedman	Friedman statistic = 8.444	
	CNO vs. Pre		*Post hoc* Dunn's		0.0537
	CNO vs. Post	Excluded the dead	*Post hoc* Dunn's		0.0228
**6d**	SUBa vs. SUBb	N = 19, 22 units	Unpaired t	Df = 39, t = 1.431	0.1603
**6c**	SUBa vs. SUBb	N = 19, 22 units	Unpaired t	Df = 39, t = 3.090	0.0037
**6g**	Sag ratio	N = 16, 9, 16, 11 neurons	One-way ANOVA	F (3, 48) = 10.4	
	A-p B vs. A-p R		*Post hoc* Turkey's		0.0423
	A-p B vs. non-A-p B		*Post hoc* Turkey's		<0.0001
	A-p B vs. non-A-p R		*Post hoc* Turkey's		0.0002
**6h**	A-p vs. non-A-p	N = 14, 17 neurons	Two-way RM ANOVA	F (1, 29) = 10.4	0.0012
**6j**	Number A-p B vs. non-A-p B	N = 16, 16 neurons	Mann-Whitney	U = 62	0.0093
	Interval A-p B vs. non-A-p B	N = 16, 16 neurons	Mann-Whitney	U = 71	0.031
**6l**	Pre vs. ZD7288	N = 8 neurons	Wilcoxon paired	W = 21	0.0313
**6m**	Pre vs. ZD7288	N = 8 neurons	Paired t	Df = 7, t = 2.4	0.0475
**7a**	SS	N = 8, 7, 8 mice	Kruskal-Wallis	Kruskal-Wallis statistic = 15.34	
	Saline vs. 20		*Post hoc* Dunn's		0.0454
	Saline vs. 50		*Post hoc* Dunn's		0.0002
	ADD	N = 8, 7, 8 mice	Kruskal-Wallis	Kruskal-Wallis statistic = 12.65	
	Saline vs. 20		*Post hoc* Dunn's		0.0515
	Saline vs. 50		*Post hoc* Dunn's		0.0009
	GSD	N = 8, 7, 8 mice	Kruskal-Wallis	Kruskal-Wallis statistic = 13.50	
	Saline vs. 20		*Post hoc* Dunn's		0.0345
	Saline vs. 50		*Post hoc* Dunn's		0.0006
**7b**	Saline vs. 50	N = 8, 8 mice	Fisher's exact		0.0014

**7d**	SS	N = 10, 9, 6 mice	Two-way RM ANOVA	F (2, 22) = 11.13	
	mCherry vs. ANT-SUB kd		*Post hoc* Scheffe's		0.0015
	EC-SUB kd vs. ANT-SUB kd		*Post hoc* Scheffe's		0.0016
	ADD	N = 10, 9, 6 mice	Two-way RM ANOVA	F (2, 22) = 6.518	
	mCherry vs. ANT-SUB kd		*Post hoc* Scheffe's		0.0071
	EC-SUB kd vs. ANT-SUB kd		*Post hoc* Scheffe's		0.0089
**7h**	Peak potential amplitude	N =7, 6, 5 mice	One-way ANOVA	F (2, 15) = 42.44	
	KD vs. KD + Light		*Post hoc* Dunnett's		<0.0001
	KD vs. KD + ZD		*Post hoc* Dunnett's		<0.0001
**7i**	fEPSP slope	N = 7, 6, 5 mice	One-way ANOVA	F (2, 15) = 22.91	
	KD vs. KD + Light		*Post hoc* Dunnett's		0.0003
	KD vs. KD + ZD		*Post hoc* Dunnett's		<0.0001
**S1d**	Sub pre vs. Sub post	N = 8, 8 EEG sections	Paired t	Df = 7, t = 3.877	0.0061
**S1e**	SS Con vs. les	N = 5, 4 mice	Two-way RM ANOVA	F (1, 7) = 14.72	0.0064
	ADD Con vs. les	N = 5, 4 mice	Two-way RM ANOVA	F (1, 7) = 14.21	0.007
**S1f**	Con vs. les	N = 5, 4 mice	Unpaired t	Df = 7, t = 3.287	0.0134
**S1g**	Con vs. les	N = 5, 4 mice	Unpaired t	Df = 7, t = 3.477	0.007
**S1i**	Con vs. les	N = 9, 9 mice	Fisher's exact		0.0023
**S1j**	SS Con vs. les	N = 9, 9 mice	Wilcoxon paired	W = 45	0.0039
	ADD Con vs. les	N = 9, 9 mice	Wilcoxon paired	W = 36	0.0078
**S3c**	SS	N = 10, 6, 7 mice	Two-way RM ANOVA	F (2, 20) = 4.945	
	EGFP vs. ChR2 Deep		*Post hoc* Scheffe's		0.005
**S3d**	ADD	N = 10, 6, 7 mice	Two-way RM ANOVA	F (2, 20) = 3.660	
	EGFP vs. ChR2 Deep		*Post hoc* Scheffe's		0.0148
**S3e**	To FS		One-way ANOVA	F (2, 20) = 14.72	
	EGFP vs. ChR2 Deep		*Post hoc* Dunnett's		0.0007
**S3f**	To sGS		One-way ANOVA	F (2, 20) = 14.72	
	EGFP vs. ChR2 Deep		*Post hoc* Dunnett's		0.0038
**S3g**	ADD	N = 5 mice	One-way RM ANOVA	F (4, 8) = 12.83	
	Pre vs. Light		*Post hoc* Dunnett's		0.0425
	Post vs. light		*Post hoc* Dunnett's		0.0176

	GSD	N = 5 mice	One-way RM ANOVA	F (4, 8) = 3.043	
	Pre vs. Light		*Post hoc* Dunnett's		0.0257
	Post vs. Light		*Post hoc* Dunnett's		0.0017
**S4d**	Deep vs. Superficial	N = 6, 7 mice	Mann-Whitney	U = 7	0.0484
**S7b**	EPSC frequency	N = 6 neurons	Friedman	Friedman statistic = 9.333	
	Pre vs. Light		*Post hoc* Dunn's		0.0078
	Post vs. Light		*Post hoc* Dunn's		0.0418
**S9b**	Control vs. KA	N = 3, 3 mice	Unpaired t	Df = 4, t = 3.629	0.0224
**S12b**	mCherry vs. KD	N = 13, 14 neurons	Unpaired t	Df = 25, t = 4.875	<0.0001
**S12c**	mCherry vs. KD	N = 13, 14 neurons	Unpaired t	Df = 25, t = 2.516	0.0187
S12e	mCherry vs. KD -55 mV	N = 13, 14 neurons	Mann-Whitney	U=59	0.1139
	mCherry vs. KD -60 mV	N = 13, 14 neurons	Mann-Whitney	U=51	0.0424
	mCherry vs. KD -65 mV	N = 13, 14 neurons	Mann-Whitney	U=49	0.0263
	mCherry vs. KD -70 mV	N = 13, 14 neurons	Mann-Whitney	U = 66	0.2005
**S13d**	Pre vs. TTA-P2	N = 10 neurons	Wilcoxon paired	W = 41	0.0156
**S13e**	SS Saline vs. TTA-P2	N = 8, 9 mice	Mann-Whitney	U = 11.5	0.0113
	GSD Saline vs. TTA-P2	N = 8, 9 mice	Mann-Whitney	U = 15	0.0426
**S14b**	SS EGFP vs. Arch	N = 7, 10 mice	Two-way RM ANOVA	F (1, 15) = 30.6	<0.0001
	ADD EGFP vs. Arch	N = 7, 10 mice	Two-way RM ANOVA	F (1, 15) = 47.69	<0.0001
**S14c**	EGFP vs. Arch	N = 7, 10 mice	Unpaired t	Df = 15, t = 3.727	0.002
**S14d**	SS	N = 8 mice	Friedman	Friedman statistic = 10	
	Pre vs. Light		*Post hoc* Dunn's		0.1542
	Post vs. Light		*Post hoc* Dunn's		0.1542
**S14e**	ADD	N = 8 mice	One-way RM ANOVA	F (8, 16) = 3.518	
	Pre vs. Light		*Post hoc* Dunnett's		0.0029
	Post vs. Light		*Post hoc* Dunnett's		0.002
**S14f**	GSD	N = 8 mice	One-way RM ANOVA	F (8, 16) = 4.219	
	Pre vs. Light		*Post hoc* Dunnett's		0.0006
	Post vs. Light		*Post hoc* Dunnett's		<0.0001
